# New composite traits for joint improvement of milk and fertility trait in Holstein dairy cow

**DOI:** 10.5713/ab.20.0600

**Published:** 2021-03-02

**Authors:** Heydar Ghiasi, Dariusz Piwczyński, Beata Sitkowska, Oscar González-Recio

**Affiliations:** 1 Payame Noor University, Faculty of Agricultural Science, Department of Animal Science, Tehran P.O.Box 19395-3697, Iran; 2 UTP University of Science and Technology, Faculty of Animal Breeding and Biology, Department of Animal Biotechnology and Genetics, Mazowiecka 28, 85-084 Bydgoszcz, Poland; 3 Institute for Agricultural and Food Research and Technology, Department of Animal Breeding, Madrid, 28040, Spain

**Keywords:** Composite Traits, Correlated Genetic Response, Days Open, Calving Interval, Milk Production, Holstein-Friesian

## Abstract

**Objective:**

The objective of this study was to define a new composite trait for Holstein dairy cows and evaluate the possibility of joint improvement in milk and fertility traits.

**Methods:**

A data set consisting 35,882 fertility related records (days open [DO], calving interval [CI], and number of services per conception [NSC], and total milk yield in each lactation [TMY]) was collected from 1998 to 2016 in Polish Holstein-Friesian breed herds. In this study TMY, DO, CI, and lactation length of each cow was used to obtain composite milk and fertility traits (CMF).

**Results:**

Moderate heritability (0.15) was estimated for composite trait that was higher than heritability of female fertility related traits: DO 0.047, CI 0.042, and NSC 0.014, and slightly lower than heritability of TMY 0.19. Favourable genetic correlations (−0.87) were estimated between CMF with TMY. Spearman rank correlation coefficients between breeding value of CMF with DO, CI, and TMY were high (>0.94) but with NSC were moderate (0.64). Selection on CMF caused favourable correlated genetic gains for DO, CI, and TMY. Different selection indices with different emphasis on fertility and milk production were constructed. The amount of correlated genetic gains obtained for DO and total milk production according to selection in CMF were higher than of genetic gains obtained for DO and TMY in selection indices with different emphasis on milk and fertility.

**Conclusion:**

The animal selection only based on a composite trait - CMF proposed in current study would simultaneously lead to favourable genetic gains for both milk and fertility related traits. In this situation CMF introduced in current study can be used to overcome to limitations of selection index and CMF could be useful for countries that have problems in recording traits, especially functional traits.

## INTRODUCTION

The unfavourable genetic correlation of fertility with milk production has been reported in Holstein dairy cows makes selection for high level of milk production or high fertility performance challenging [1–3]. In the recent research of Yamazaki et al [4] the genetic correlations between fertility traits were strong, whereas those among fertility traits and lactation persistency were weak and undesirable. Genome-wide association analysis revealed that there are several single nucleotide polymorphisms (SNPs) having favourable effects on both milk yield and some fertility traits such as days open and interval from first to last insemination [5]. Several SNPs associated with daughter pregnancy rate were identified that were not negatively associated with production traits, hence it is conceivable to improve daughter pregnancy rate without a side effect on production traits [6]. Therefore from the genetic point of view concurrent improvement of production traits and fertility performance may be possible by focusing on a few relevant SNPs [7]. The antagonistic correlation between milk trait and fertility is not unity which implies it is possible to find sires that are best for both milk yield and fertility and therefore fertility and milk production can be improved simultaneously [8]. Nowadays most countries have integrated fertility in total merit selection indices that decelerated the degradation of fertility traits [9–11]. In Poland, from 2007 for bulls, and from 2014 for cows in breeding work, the index “Production and Functionality” has been used [11]. The index formula is focused on improving production (weight = 0.40) and functional traits covering fertility (weight = 0.15), longevity (weight = 0.10), conformation (weight = 0.15), and somatic cells (weight = 0.10). But some studies show that including fertility along with milk production in a breeding programme may not produce a favourable genetic response in fertility traits [12,13].

To overcome adverse genetic correlation in breeding programmes some strategies have been proposed. For example, genetic antagonism between milk yield and somatic cell count has been reported [14]. Moxley et al [15] concluded that as milking managements improve, somatic cell count decreases and milk production increases. The other methods to deal with joint improvement of traits with antagonistic correlation are using linear programming [16], desired-gain index [17], and restricted selection index [18]. Ghiasi et al [13] show it is possible to simultaneously improve milk production and fertility performance in Holstein cow using proportional restricted selection index, although unfavourable genetic correlation exists between milk production and fertility. Composite trait definition is the other method to deal with unfavourable genetic correlation in breeding programmes. This method is widely used in sheep breeding programmes to jointly improve reproductive performance and productivity traits [19]. Reproductive and production traits in sheep are complex traits influenced by several component traits such as, ovulation rate, embryo survival, number of lambs born, lamb survival, lactation and lamb growth, and adverse genetic correlations have been reported between these component traits [20]. Litter weight weaned per ewe is a composite trait whose selection based on this composite trait can result in favourable genetic response in all component traits including ewe fertility performance, lamb survival and lamb growth rate [21]. In dairy cattle breeding programmes description of novel traits in the United States and Canada was presented in research paper by Chesnais et al [22] and these include, for example: new predictors of mastitis incidence, milk composition (e.g., MIR) or profit per cow.

The objective of this study was to define a new composite trait for Holstein dairy cows and evaluate the possibility of joint improvement in milk and fertility trait using this new composite trait.

## MATERIALS AND METHODS

### Data

A data set consisting 35,882 fertility related records (days open [DO], calving interval [CI], number of services per conception [NSC], and total milk yield in each lactation [TMY]) was collected from 1998 to 2016 for to 23,160 Polish Holstein-Friesian breed cows in 40 herds (Table 1). The number of records considered in subsequent years fluctuated below 1,000 until 2004. From 2005 to 2016 the number of records were recorded between 1,500 and 3,500.

In this study TMY, DO, CI, and lactation length (LL) of each cow was used to obtain composite milk and fertility traits (CMF) for each cow as follows:

(1) $$\text{CMF}=\left[\frac{\left(\text{TMY}\right)}{\left(\text{CI}\right)}\times \frac{\left(305-\text{DO}\right)}{\left(\text{LL}\right)}\right]$$

### Description of the composite milk and fertility traits

The section of $\frac{\left(\text{TMY}\right)}{\left(\text{CI}\right)}$ in the CMF means cows that have high milk production in each lactation and low CI will have high value for CMF, then this value will be adjusted by $\frac{\left(305-\text{DO}\right)}{\left(\text{LL}\right)}$.

The objective of including section $\frac{\left(305-\text{DO}\right)}{\left(\text{LL}\right)}$ in the CMF was to find cows that have lower days open and produce more milk in smaller LL.

### Statistical analysis

The variance components for CMF, TMY, DO, and CI were estimated using the following statistical model:

(2) $$\text{y}={\text{X}}_{\text{b}}+{\text{Z}}_{\text{a}}+{\text{W}}_{\text{pe}}+\text{e}$$

y, traits; b, fixed effects (herd, parity, season of calving, year of calving); a, additive genetic variance; pe, permanent environmental effect; e, residual effect for the traits; X, Z, W, incidence matrices relating observations to effects. ASREML software [23] was used to estimate genetic parameters.

### Genetic gains

The correlated genetic gains in DO, CI, NSC, and TMY (Δ*G_x_*) to selection for CMF were calculated as:

(3) $$\Delta G_{x}=\frac{r_{G}\times r_{CMF}\times i_{CMF}\times \delta_{Ax}}{L}$$

r_G_, additive genetic correlation between CMF with trait x; r_CMF_, accuracy of trait CMF; i_x_, selection intensity of trait CMF; σ_Ax_, additive genetic standard deviation for trait x and L, generation interval. In current study the values of σ_Ax_ and r_G_ were illustrated in Table 2 and 3; r_CMF_ was 0.47; and I was equal to one in this study.

Different selection indices were constructed to compare genetic gains obtained based on selection on CMF with genetic gains obtained based on selection on indices with different emphasis on production and fertility traits. DO as fertility trait and TMY were considered in breeding goal (H).

(4) $$\text{H}={\text{w}}_{1}\times \text{TMY}+{\text{w}}_{2}\times \text{DO}$$

(5) $${\text{I}}_{1}={\text{b}}_{1}\times \text{TMY}+{\text{b}}_{2}\times \text{DO}$$

Selection indices weights were derived with considering following relative emphasis on milk production and fertility (50:50, 60:40, 70:30, and 80:20). For example, to obtain relative emphasis of milk to fertility of 60:40 the w_1_ and w_2_ were calculated for the following fraction to be 60 to 40.

Relative emphasis of TMY to trait where:

σ_A_TMY__ and σ_A_DO__ additive genetic standard deviation of TMY and DO; w_1_ and w_2_ index weights for TMY and DO.

Expected genetic gains for those traits in the selection index (ΔG_j_) was predicted as follows:

(6) $$\Delta {\text{G}}_{\text{j}}=({{\text{G}}_{\text{j}}}^{\prime }\text{b}/\sigma_{\text{I}})\times \text{i}$$

Where, G_j_, *j*th column of the matrix G related to *j*th trait in aggregate genotype; σ_I_, standard deviation of the selection index calculated as $\sigma_{\text{I}}=\sqrt{\left({\text{b}}^{\prime }\text{P}\;\text{b}\right)}$; i, selection intensity.

b, index weighs that were w_1_ and w_2_ because traits in index and breeding goal are same. In this study the generation interval and selection intensity were set to one to simplify comparison.

## RESULTS AND DISCUSSION

The most important goal of dairy farmers is to get as much milk as possible from the cows’ ration maintained for each day of the calendar year. The presently proposed composite trait CMF formula allows this goal to be achieved in two ways: by using cows with short lactations, which quickly reach the peak of lactation, or by using cows that maintain high production throughout the whole lactation, at the expense of prolonged CI and DO. The latter strategy has the advantage of reducing the farmer’s costs associated with inseminating cows and supervising delivery. It should be emphasised that the CI, DO, NSC features included in the CMF formula represent the most important fertility features, routinely included in many national dairy cattle programmes [9]. As indicated by the results of numerous studies, CI and DO are very strongly correlated [24]. However, including both these traits in the CMF formula was necessary and fully justified, as the reproductive information that they provide complement each other. Their values are a consequence of proper heat identification, the number of insemination procedures performed, the time of service, and postpartum downtime.

### Genetic parameters

Variance component and heritability of CMF, DO, CI, NSC, and TMY are illustrated in Table 2. Heritability estimates for TMY were moderate (0.19) and heritability of fertility related traits (DO, CI, NSC) was low (0.014 to 0.047). The estimated heritability for CMF (0.15) was higher than heritability of female fertility related traits (DO, NSC, and CI) and slightly lower than heritability of TMY.

These estimates agree with the results obtained by Wall et al [25], González-Recio et al [12], Ghiasi et al [13].

The favourable genetic correlations were estimated between CMF with milk and fertility traits (Table 3). The estimated genetic correlation between CMF with TMY was strong and positive, which means selecting animals with high value for CMF will directly cause high milk yield. At the same time, a strong (CI, DO) and moderate (NSC) negative genetic correlation was found between CMF and fertility traits, which means that selecting animals with a high CMF value should translate into the shorter DO, CI, and lower NSC. These favourable genetic correlations of CMF with milk and fertility traits indicate that selection of animals according to proposed composite trait CMF will cause simultaneous genetic improvement in milk and fertility performance.

The moderate heritability obtained for CMF compared to low heritability obtained for fertility traits, and at the same time strong genetic relationships between these traits indicates selection based on CMF would genetically improve the fertility performance faster than selection based on fertility traits (DO, CI, NSC). Moderate, positive genetic correlations between TMY and fertility were estimated in the studies, which clearly indicate that direct selection only for milk yield will adversely affect CI, DO, and increase number of NSC. Studies show that selection only based on milk production will cause undesirable genetic gains for fertility traits [12,13]. Unfavourable genetic correlations between fertility traits and TMY and beneficial ones from CMF clearly prove that the proposed composite trait (CMF) can have practical applications for improving dairy cattle.

### Genetic responses

The correlated genetic responses obtained for DO, CI, NSC, and TMY based on selection in CMF are shown in Table 4. Also, genetic gains achieved for DO and TMY according to selection indices with different emphasis are illustrated in Table 5. Considerable favourably correlated genetic gains were obtained for DO, CI, and TMY by selection on CMF. Amount of genetic gains for DO and CI was nearly the same and were −5.21 and −5.25 days, respectively. The amount of genetic gains for NSC and TMY was −0.024 and 525 kg, respectively. Genetic gains obtained for DO and TMY in selection indices with different emphasis were lower than genetic gains obtained for DO and TMY in selection on CMF. When emphasis on milk in selection indices increased from 50 to 80, the amount of genetic gains for TMY increased from 89.75 kg to 139.03 kg but when emphasis on fertility decreased from 50 to 20 in selection indices the amount of genetic gains for DO almost was constant and ranged from 0.88 to 0.95 days per generation. The results of this study show that using CMF for genetic evolution simultaneously will improve the DO and TMY and the amount of genetic gains will be higher than when using selection indices with different emphasis on fertility and milk production. The introduced composite trait (CMF) simultaneously improved fertility and milk yield without constructing a selection index which, as we know, involves some problems such as the need to calculate economic value and the economic situation will be different between countries.

### Spearman rank correlation

Spearman rank correlation coefficients between breeding value of CMF with fertility and milk traits are shown in Table 6. The strongest correlations were obtained between CMF with DO, CI, and TMY. These results indicate that ranking animals by breeding value estimated based on CMF will be similar to ranking animals based on breeding values based on DO, CI, and TMY. It should be noted that analogous genetic correlations with similar directions and power of dependence estimated in current study were reported in other studies [24,25]. Therefore, selection of animals according to only milk production will cause an unfavourable genetic response for DO, CI, NSC, and conversely. But CMF is a new trait that defines cows that have high milk production in each lactation, low CI and low days open. High favourable genetic correlation and high Spearman rank correlation were obtained between CMF with DO, CI, and TMY. Therefore, animal selection only based on CMF would simultaneously lead to favourable genetic gains for both milk and fertility related traits (DO, CI, and NSC) although there is unfavourable genetic correlation between milk production and fertility traits. The proportional restricted selection index theory [26] has been proposed to simultaneously improve traits that have unfavourable genetic correlations but using this selection index requires estimation of breeding value and economic value for each trait and also the amount of genetic gains obtained for each trait will be lower than genetic gains obtained when selection is based on only single traits [13]. But using the CMF as new trait for genetic evaluation to jointly improve fertility and milk production can overcome the limitations mentioned in using proportional restricted selection. The other advantage of using CMF to obtain favourable genetic gain in both milk and fertility is that the heritability of CMF compared to heritability of fertility traits is high (3.75 to 10.7 times) even though the heritability of CMF is a little bit lower than heritability of milk production (0.15 vs 0.19).

Some composite traits have been introduced in dairy cow such as longevity, residual feed intake (RFI), udder composite, net merit, feet and legs composite. The RFI is introduced as composite trait to select for enhanced feed efficiency [27,28]. Studies in beef cattle show selection for reducing RFI should reduce animal feed intake, greenhouse gas emission and nutrient loss in manure without negative impact on production traits such as milk production [29]. Compared to above mentioned composite traits in dairy cow a CMF is a composite trait that considers many traits such as: traits related to milk production, fertility, mastitis, disease, and all traits influencing milk and fertility. Many countries have constructed national selection index that includes production and functional traits [11,30]. Constructing selection index has its limitation such as: parameters of traits changing due to selection and problems related to estimating relative economic value [30].

## CONCLUSION

The results of this study show animal selection only based on a composite trait—CMF proposed in current study would simultaneously lead to favourable genetic gains for both milk and fertility related traits. In this situation CMF introduced in current study can be used to overcome to limitations of selection index and CMF could be useful for countries that have problems in recording traits, especially functional traits. The proposed trait CMF characterized by comparable heritability to MY, several times greater than DO and CI may be of practical use in herds that are not covered by the official breeding value assessment, it creates the possibility of effective selection of cows in the above herds, even when it is carried out on the basis of the calculated CMF (phenotypic value).

## Figures and Tables

**Table 1:** Descriptive statistics of data used for analysis

Trait	No. of records	Mean	Standard deviation
TMY	35,882	11,069	2,945
CI	35,882	412	66
DO	35,882	133	66
NSC	35,882	1.97	1.42

TMY, total milk yield in each lactation (kg); CI, calving interval (d); DO, days open (d); NSC, number of services per conception (no.).

**Table 2:** Genetic parameters for composite milk and fertility traits, total milk yield in each lactation, calving interval, days open and number of services per conception

Traits	Additive genetic	Permanent environmental	Residual	Heritability

	Variance			
CMF	10.45	3.89	55.31	0.150±0.010
TMY	1,655,830	3,306,470	3,745,710	0.190±0.013
CI	181.95	305.20	3,786.48	0.042±0.007
DO	188.03	489.44	3,300.69	0.047±0.008
NSC	0.028	0.206	1.75	0.014±0.005

CMF, composite milk and fertility traits; TMY, total milk yield in each lactation (kg); CI, calving interval (d); DO, days open (d); NSC, number of services per conception (no.).

**Table 3:** Genetic and phenotypic correlations between new composite milk and fertility traits, milk production and fertility traits

Traits	Genetic correlation	Phenotypic correlation
CMF		
TMY	0.87±0.031	0.15±0.005
CI	−0.83±0.0017	−0.84±0.038
DO	−0.81±0.04	−0.83±0.001
NSC	−0.32±0.12	−0.01±0.005
TMY		
CI	0.55	
DO	0.50	
NSC	0.44	

CMF, composite milk and fertility traits; CI, calving interval (d); DO, days open (d); NSC, number of services per conception (no.); TMY, total milk yield in each lactation (kg).

**Table 4:** Correlated genetic responses for total milk yield in each lactation, calving interval, days open and number of services per conception in selection for composite milk and fertility traits

Traits	Correlated genetic responses per generation for CMF
TMY	525
CI	−5.25
DO	−5.21
NSC	−0.024

CMF, composite milk and fertility traits; TMY, total milk yield in each lactation (kg); CI, calving interval (d); DO, days open (d); NSC, number of services per conception (no.).

**Table 5:** Selection indices with different relative emphasis and genetic responses per generation

Relative emphasis of milk to fertility	Selection index	Genetic responses per generation	

		TMY	DO
50:50	I_1_ = (1×TMY)+(93.80×DO)	89.75	0.95
60:40	I_1_ = (1×TMY)+(55.30×DO)	103.17	0.89
70:30	I_1_ = (1×TMY)+(40.22×DO)	114.36	0.88
80:20	I_1_ = (1×TMY)+(23.54×DO)	139.03	0.90

TMY, total milk yield in each lactation (kg); DO, days open (d).

**Table 6:** Spearman rank correlation between breeding value of animal for composite milk and fertility traits with breeding value of animal with total milk yield in each lactation, calving interval, days open and number of services per conception

Item	Traits	Spearman rank correlation
BV	CMF-TMY	0.97
BV	CMF-CI	0.96
BV	CMF-DO	0.94
BV	CMF-NSC	0.64

BV, breeding value; CMF, composite milk, and fertility traits; TMY, total milk yield in each lactation (kg); CI, calving interval (d); DO, days open (d); NSC, number of services per conception (no.).
